# TLR7/9-mediated mucosal innate immune dysregulation in IgA nephropathy

**DOI:** 10.3389/fimmu.2026.1843019

**Published:** 2026-07-08

**Authors:** Mingfeng Lee, Hitoshi Suzuki, Yuko Makita, Yusuke Suzuki

**Affiliations:** 1 Department of Nephrology, Juntendo University Faculty of Medicine, Tokyo, Japan; 2 Department of Nephrology, Juntendo University Urayasu Hospital, Chiba, Japan

**Keywords:** galactose-deficient IgA1, IgA nephropathy, mucosal immunity, toll-like receptor 7, toll-like receptor 9

## Abstract

IgA nephropathy (IgAN) is the most common primary glomerulonephritis worldwide and is characterized by mesangial deposition of nephritogenic IgA-containing immune complexes. Increasing evidence suggests that dysregulated mucosal innate immune responses contribute to disease pathogenesis, particularly through activation of endosomal Toll-like receptors (TLRs). Among these, TLR7 and TLR9, which recognize single-stranded RNA and unmethylated CpG DNA, respectively, have been implicated in the abnormal mucosal immune activation observed in IgAN. Experimental and clinical studies have demonstrated increased expression of TLR7 and TLR9 in mucosal tissues, including the tonsils of patients with IgAN. Activation of these pathways may promote galactose-deficient IgA1 (Gd-IgA1) production through interleukin-6 (IL-6)- and APRIL-mediated mechanisms, thereby contributing to the formation of nephritogenic immune complexes. IgAN-prone mouse studies further support the role of TLR-mediated immune activation in mesangial IgA deposition and glomerular injury, although limitations remain in fully recapitulating human disease. Recent humanized mouse models further suggest that Gd-IgA1 alone may be insufficient to induce disease, highlighting the importance of mucosal immune context in shaping nephritogenic IgA responses. In addition, emerging genetic and translational studies suggest potential links between TLR signaling pathways and susceptibility to IgAN. Recent therapeutic advances targeting mucosal immunity and related downstream pathways, including hydroxychloroquine and APRIL/BAFF-directed therapies, have further highlighted the clinical relevance of innate immune dysregulation in IgAN. In this review, we summarize current evidence regarding the roles of TLR7 and TLR9 in IgAN, with particular emphasis on their contribution to mucosal immune abnormalities and their potential therapeutic implications.

## Introduction

IgA nephropathy (IgAN), first described by Berger over half a century ago, is characterized by mesangial deposition of IgA-containing immune complexes. Despite its relatively indolent clinical course, up to 40% of untreated patients progress to end-stage kidney disease within two decades. Although substantial therapeutic advances have been made in recent years, no curative therapy has yet been established, underscoring the need to better understand the pathogenic mechanisms underlying the disease and to identify novel therapeutic targets. IgAN is the most common primary glomerulonephritis worldwide, with the highest prevalence observed in East Asia, followed by Europe, whereas the incidence is lower in Africa and North America (1). As a major cause of kidney failure in young adults, IgAN remains an important global health concern.

The current understanding of IgAN pathogenesis is largely based on the multi-hit hypothesis, which involves the overproduction of galactose-deficient IgA1 (Gd-IgA1), the generation of antiglycan autoantibodies, formation of circulating immune complexes, and mesangial deposition leading to glomerular injury (2). Among these steps, accumulating evidence suggests that dysregulated mucosal immunity plays an important role in initiating abnormal IgA responses.

Recent studies have increasingly implicated innate immune activation mediated by endosomal Toll-like receptors (TLRs), particularly TLR7 and TLR9, in the pathogenesis of IgAN (3–6). TLR7 recognizes single-stranded RNA, whereas TLR9 senses unmethylated CpG DNA, and both receptors are expressed in dendritic cells, macrophages, and B cells within mucosal immune tissues. Experimental and clinical studies have demonstrated enhanced expression and activation of these pathways in patients with IgAN, suggesting their potential contribution to abnormal Gd-IgA1 production and nephritogenic immune responses. However, the extent to which these pathways directly drive human disease remains incompletely understood.

In this review, we focus on the roles of TLR7 and TLR9 in mucosal innate immune dysregulation in IgAN, integrating evidence from experimental models, genetic studies, and clinical observations. We further discuss the therapeutic implications and current limitations of targeting TLR-related pathways in IgAN.

## Mucosal innate immunity and endosomal TLRs in IgAN

Aberrant mucosal immune responses have long been implicated in the pathogenesis of IgAN (7, 8). Clinically, episodes of gross hematuria frequently occur following upper respiratory tract infections such as tonsillitis, supporting the concept that mucosal immune dysregulation contributes to disease activity. A central feature of IgAN is the overproduction of Gd-IgA1, an abnormal IgA1 isoform characterized by deficient O-glycosylation within its hinge region. Elevated circulating levels of Gd-IgA1 are consistently observed in patients with IgAN and represent a key initiating factor in the multi-hit model of disease pathogenesis (9). Gd-IgA1 is recognized by antiglycan autoantibodies, leading to the formation of nephritogenic immune complexes that subsequently deposit within the glomerular mesangium (2).

Mucosal lymphoid tissues, including the tonsils and intestinal Peyer’s patches, are considered important sites of pathogenic IgA production (7, 8). Supporting this concept, tonsillectomy has been associated with reductions in circulating Gd-IgA1 levels and improvement of urinary abnormalities in some patients with IgAN (10). Although no disease-specific microbial antigen has been definitively identified, several microbial species have been proposed as potential triggers of mucosal immune activation. For example, oral commensals such as cnm-positive Streptococcus mutans and Campylobacter rectus have been associated with renal dysfunction and disease severity in IgAN (11, 12). A recent study further demonstrated an association between cnm-positive S. mutans and glomerular deposition of Gd-IgA1, supporting a potential link between oral microbial dysbiosis and pathogenic IgA responses in IgAN (13). These observations support the hypothesis that chronic mucosal exposure to microbial components may contribute to sustained innate immune activation.

Recent evidence has further highlighted the importance of mucosal infections and gut microbiota in shaping host immune responses in IgAN (14). Alterations in the intestinal microbiome may increase antigenic stimulation and promote aberrant mucosal innate immune activation, thereby promoting the production of a proliferation-inducing ligand (APRIL) and B-cell activating factor (BAFF), leading to enhanced IgA class switching. Experimental and clinical observations collectively support the concept that chronic exposure to microbial components may contribute to dysregulated IgA responses in IgAN (4, 15, 16).

Among innate immune pathways, increasing attention has focused on endosomal TLRs, particularly TLR7 and TLR9. Importantly, tonsillar tissues from patients with IgAN exhibit increased expression of TLR7 and TLR9 compared with controls with chronic tonsillitis (3, 6). Enhanced TLR9 expression has been associated with favorable responses to tonsillectomy, including reductions in serum Gd-IgA1 levels and remission of hematuria (17, 18). In addition, increased expression of TLR7 and TLR9 in tonsillar tissues has been associated with enhanced APRIL expression, supporting a relationship between endosomal TLR activation and pathogenic B-cell responses in IgAN (3, 6).

Collectively, these findings suggest that dysregulated mucosal innate immunity mediated by endosomal TLRs may contribute to abnormal IgA responses in IgAN. However, most available evidence remains associative, and the precise mechanisms linking TLR activation to human disease pathogenesis require further clarification.

## TLR7/9-mediated mechanisms of Gd-IgA1 production

The current understanding of IgAN pathogenesis is largely based on the multi-hit hypothesis, in which the overproduction of Gd-IgA1 represents the initiating step leading to the formation of nephritogenic immune complexes and subsequent glomerular injury (2). TLR7 and TLR9 have been proposed as important upstream regulators of Gd-IgA1 production through their effects on cytokine production, B-cell activation, and IgA-producing cell responses.

In IgAN, tonsillar B cells have been shown to express TLR7 and TLR9, whereas the expression of other nucleotide-sensing TLRs, such as TLR3 and TLR8, appears to be limited (19, 20). These findings suggest that TLR7 and TLR9 may play particularly important roles in mucosal immune activation associated with IgAN.

Among downstream mediators, interleukin-6 (IL-6) has emerged as a key cytokine linking TLR activation to abnormal IgA glycosylation. Experimental studies have demonstrated that IL-6 downregulates core 1 β1, 3-galactosyltransferase (C1GALT1), a glycosyltransferase required for normal O-glycosylation of IgA1, thereby promoting the production of Gd-IgA1 (21). In parallel, activation of TLR-mediated innate immune pathways induces the production of APRIL and BAFF, which promote IgA class-switch recombination, plasma cell survival, and sustained IgA production (22, 23). Recent studies have further integrated these findings, highlighting a mechanistic link between TLR7/9 activation, APRIL/BAFF signaling, and pathogenic Gd-IgA1 production in tonsillar B cells, thereby reinforcing the importance of mucosal innate immune dysregulation in IgAN pathogenesis (16).

Recent evidence has further expanded this concept by identifying plasmacytoid dendritic cells (pDCs) as potential contributors to TLR9-mediated Gd-IgA1 production. In patients with IgAN, tonsillar pDC abundance correlated with TLR9 and APRIL expression, while depletion of pDCs reduced aberrantly-glycosylated IgA production in both human tonsillar cell cultures and CpG-stimulated ddY mice, supporting a role for the TLR9–APRIL axis in nephritogenic IgA generation (24).

Experimental studies further support the relationship between TLR activation and nephritogenic IgA responses. Stimulation with CpG oligodeoxynucleotides (TLR9 agonists) or imiquimod (TLR7 agonist) enhances serum IgA levels, aberrantly-glycosylated IgA production, and immune complex formation in murine models of IgAN (4, 6, 25). These findings suggest that TLR7/9-mediated mucosal innate immune activation may contribute to the amplification of pathogenic IgA responses. However, the relative contribution of these pathways in human disease remains incompletely understood, and most available human data remain associative rather than directly causal.

**Figure 1:**
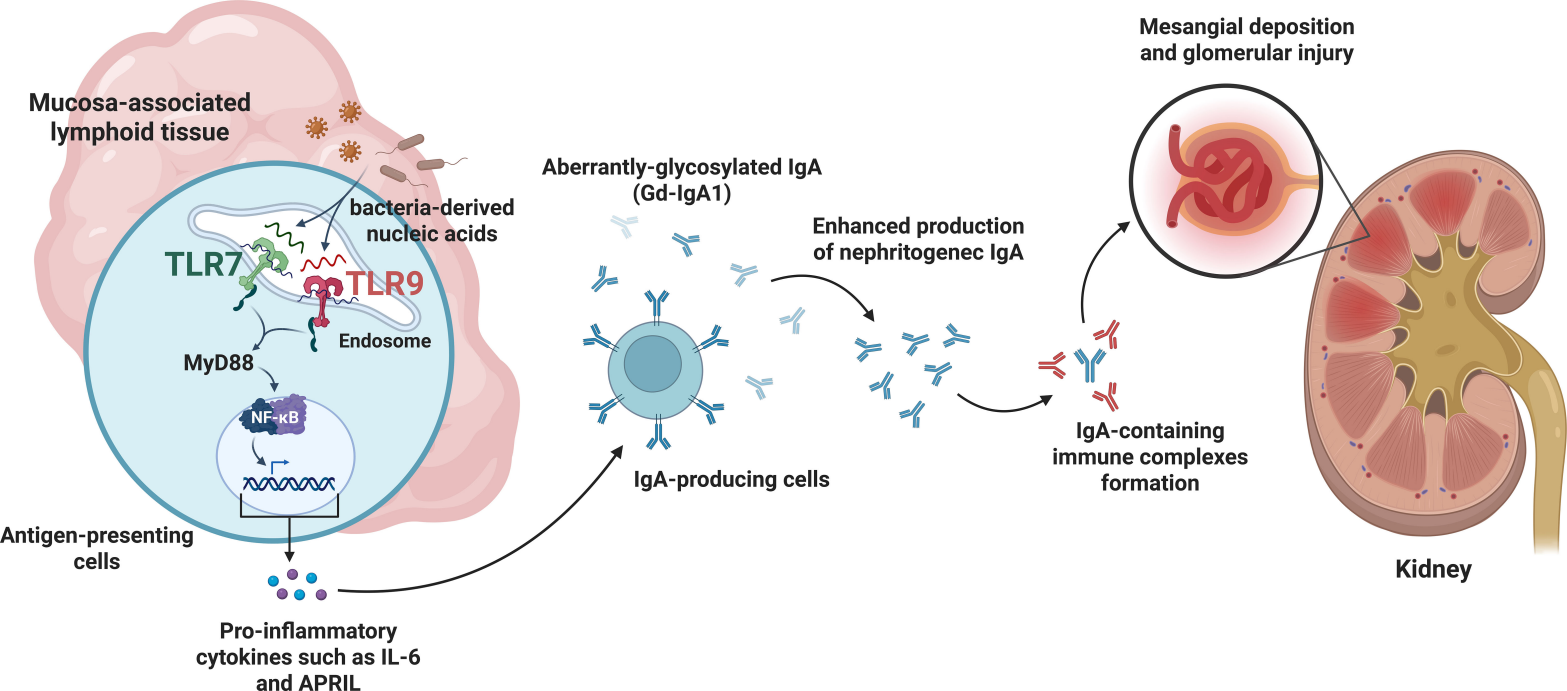
Proposed mechanisms by which TLR7- and TLR9-mediated mucosal innate immune activation may contribute to IgA nephropathy (IgAN) pathogenesis. Microbial or viral nucleic acids activate endosomal TLR7 and TLR9 in mucosal immune cells, leading to MyD88-dependent signaling and the production of pro-inflammatory mediators such as interleukin-6 (IL-6) and a proliferation-inducing ligand (APRIL). These pathways may promote the production of galactose-deficient IgA1 (Gd-IgA1) and nephritogenic immune complexes, contributing to mesangial deposition and glomerular injury in IgAN. This figure was created with BioRender.com.

Taken together, current evidence supports a model in which TLR7/9-mediated mucosal innate immune activation contributes to abnormal IgA glycosylation and enhanced nephritogenic IgA production through IL-6- and APRIL-associated pathways (**Figure 1**). These mechanisms may represent an important link between mucosal immune dysregulation and systemic disease manifestations in IgAN.

## Evidence from murine models

Murine models have provided important insights into the potential role of TLR-mediated innate immune activation in IgAN, although no single model fully recapitulates the complexity of human disease. These experimental systems have nevertheless been instrumental in elucidating mechanisms linking mucosal immune activation to nephritogenic IgA production.

Among the most extensively studied models are ddY mice, which spontaneously develop mesangial IgA deposition and glomerular injury resembling human IgAN (26). Because disease severity varies substantially among individual ddY mice, grouped ddY (gddY) strains have been established to provide a more homogeneous and accelerated disease phenotype (27). Genetic studies have identified MyD88, a central adaptor molecule downstream of TLR signaling, as a susceptibility locus associated with severe nephropathy (28). Furthermore, experimental activation of TLR7 or TLR9 exacerbates nephritis and enhances pathogenic IgA responses in these models (4, 6, 25), supporting a role for endosomal TLR activation in disease progression.

Additional insights have been obtained from humanized IgA1 models (29). Because mice do not naturally express human IgA1 or its hinge-region O-glycans, these systems permit investigation of Gd-IgA1, a key component of the multi-hit hypothesis. Humanized α1KI/CD89 transgenic mice develop mesangial IgA deposition, hematuria, proteinuria, complement activation, and progressive renal injury (30), thereby serving as a useful translational model for investigating IgA1-specific pathogenic mechanisms and evaluating novel therapeutic strategies. However, these models rely on artificial transgene expression and therefore reproduce only selected aspects of human disease.

More recently, a humanized IgA1 knock-in mouse with B-cell-specific deletion of C1galt1, the enzyme responsible for galactosylation of IgA1 O-glycans, was developed (31). Despite markedly elevated circulating Gd-IgA1 levels, these mice exhibited minimal mesangial IgA deposition under both physiological and inflammatory conditions. Furthermore, mucosa-derived IgA1 induced substantially greater mesangial deposition than serum- or myeloma-derived IgA1, despite comparable or even lower Gd-IgA1 content. These findings challenge the notion that Gd-IgA1 alone is sufficient to drive disease and instead suggest that the tissue origin and immune context of IgA production are critical determinants of pathogenicity (31).

From the perspective of TLR biology, these observations are particularly noteworthy because they further support the importance of mucosal immune activation in IgAN pathogenesis. Rather than identifying Gd-IgA1 as an isolated pathogenic factor, this model emphasizes the contribution of the mucosal immune environment in shaping nephritogenic IgA responses. This concept is consistent with accumulating evidence linking TLR7/9-mediated mucosal innate immune activation to abnormal IgA production and disease progression.

Each model provides distinct insights into IgAN pathogenesis. gddY mice are particularly useful for investigating mucosal innate immunity and TLR7/9-mediated disease mechanisms, whereas humanized IgA1 models facilitate the study of aberrant IgA1 glycosylation and immune complex formation. More recent B-cell-specific C1galt1 knockout models directly examine the pathogenic significance of Gd-IgA1. However, no single model fully reproduces all features of human IgAN, highlighting the need for complementary experimental approaches when interpreting mechanistic findings.

Taken together, murine models support the concept that TLR7/9-mediated mucosal innate immune activation contributes to nephritogenic IgA responses. However, species-specific differences in IgA biology, the absence of a perfect murine equivalent of human IgAN, and variability among experimental systems necessitate caution when extrapolating findings to human disease.

## Genetic and clinical evidence linking TLR pathways to IgAN

Accumulating genetic and clinical evidence supports a potential role for TLR-mediated innate immune activation in IgAN, although direct causal relationships remain difficult to establish in humans.

Among TLR-related genetic factors, polymorphisms within the TLR9 gene have been most extensively investigated. Several studies have reported associations between TLR9 variants and disease susceptibility, clinical severity, and histological progression in IgAN. In particular, the rs352140 polymorphism has been associated with disease severity, tonsillar TLR9 expression, and responses to tonsillectomy-based therapy in patients with IgAN (17, 28). Although the functional consequences of individual variants remain incompletely understood, these findings suggest that genetic variation in TLR signaling pathways may influence disease expression.

Genome-wide association studies (GWAS) have also identified multiple susceptibility loci related to IgA production and its regulation, including TNFSF13 (encoding APRIL) and C1GALT1 (32–35). These loci provide additional support for the concept that dysregulated mucosal immune activation and abnormal IgA glycosylation contribute to disease pathogenesis.

Beyond these susceptibility loci, recent fine-mapping studies have identified MTMR3 as a potential causal gene within the MTMR3/HORMAD2/LIF/OSM risk locus. Functional analyses demonstrated that MTMR3 promotes IgA production, whereas Mtmr3-deficient mice exhibited impaired TLR9-induced IgA responses, reduced glomerular IgA deposition, and attenuated mesangial cell proliferation (36). These findings suggest that genetic factors regulating TLR9-mediated mucosal immunity may contribute to disease susceptibility and progression in IgAN.

Interestingly, the prevalence of risk alleles and the strength of genetic associations vary among ethnic groups, with stronger genetic signals generally observed in East Asian populations than in European populations (37). These observations suggest that host genetic background may influence the relative contribution of TLR-mediated pathways to disease susceptibility and progression.

Clinical studies further support the involvement of TLR-mediated mucosal immune activation. Increased expression of TLR7 and TLR9 has been demonstrated in tonsillar B cells and antigen-presenting cells from patients with IgAN (3, 6). Moreover, elevated TLR expression has been associated with enhanced APRIL production, increased Gd-IgA1 levels, and disease activity. Episodes of gross hematuria frequently occur following upper respiratory tract infections, supporting a link between mucosal immune stimulation and disease exacerbation.

Clinical observations following mRNA vaccination have also provided additional insights into the potential role of innate immune activation in IgAN (38–40). Although the precise mechanisms remain uncertain, activation of innate immune pathways, including nucleic acid-sensing TLRs, has been proposed as a potential contributing factor (41). These observations provide additional clinical support for the concept that mucosal and innate immune activation may influence disease activity in susceptible individuals.

Despite these findings, important limitations should be acknowledged. Most available human studies are observational and demonstrate association rather than causation. Furthermore, genetic associations generally exhibit modest effect sizes and have not been consistently replicated across all populations. Therefore, while current genetic and clinical evidence supports the involvement of TLR pathways in IgAN, the precise contribution of TLR7/9 signaling to disease initiation and progression remains to be fully elucidated.

## Therapeutic implications of TLR-related pathways in IgAN

Increasing recognition of the role of TLR-mediated mucosal innate immune activation in IgAN has generated interest in therapeutic strategies targeting these pathways. Although no treatment specifically targeting TLR7 or TLR9 has yet been approved for IgAN, several currently available or emerging therapies may modulate TLR-related pathogenic mechanisms.

Hydroxychloroquine (HCQ), an inhibitor of endosomal acidification, suppresses signaling through nucleic acid-sensing TLRs, including TLR7 and TLR9 (42). Clinical studies have demonstrated that HCQ reduces proteinuria in patients with IgAN (43, 44), while experimental studies have shown attenuation of nephritogenic IgA responses and glomerular injury (6). These findings support the concept that modulation of endosomal TLR signaling may represent a therapeutic strategy in selected patients.

In addition to direct modulation of TLR-related pathways, increasing attention has focused on downstream mediators of mucosal innate immune activation. APRIL and BAFF play central roles in B-cell survival, plasma-cell differentiation, and IgA production (22, 23). Several biologic agents targeting APRIL or dual APRIL/BAFF signaling have demonstrated promising results in clinical trials, including reductions in proteinuria and circulating Gd-IgA1 levels (45–48). Although these therapies do not directly inhibit TLR signaling, they target pathways that may be activated downstream of TLR7/9-mediated immune responses.

Targeting mucosal immune activation has also emerged as a promising therapeutic approach. Nefecon, a targeted-release formulation of budesonide designed to act on Peyer’s patches, reduces proteinuria and slows kidney function decline in patients with IgAN (49–51). While its mechanism of action is not specific to TLR pathways, the clinical efficacy of mucosal-targeted therapy further supports the importance of mucosal immune dysregulation in disease pathogenesis.

Despite these advances, the precise therapeutic value of directly targeting TLR7 or TLR9 remains uncertain. Future studies are needed to determine whether selective inhibition of endosomal TLR signaling can provide clinical benefit while preserving protective host immune responses. Improved understanding of TLR-mediated mucosal immunity may facilitate the development of more precise and mechanism-based therapies for IgAN.

## Conclusion

Accumulating evidence supports the involvement of endosomal TLRs, particularly TLR7 and TLR9, in the mucosal innate immune dysregulation associated with IgAN. Experimental studies have shown that activation of these pathways can promote IL-6- and APRIL-mediated IgA responses, enhance production of nephritogenic IgA, and exacerbate glomerular injury. Human studies, including analyses of tonsillar tissues, genetic associations, and clinical observations, further suggest that TLR-related pathways may contribute to disease activity in susceptible individuals.

However, the precise contribution of TLR7/9 signaling to human IgAN remains incompletely defined. Current evidence supports their role as important contributors to mucosal immune activation, rather than as established dominant drivers of disease. Recent murine studies further emphasize that Gd-IgA1 alone may be insufficient to induce IgAN-like pathology, highlighting the importance of tissue origin, immune context, and mucosal immune environment in shaping pathogenic IgA responses.

Future studies integrating experimental models, human mucosal tissue analyses, genetic data, and clinical biomarkers will be essential to clarify the role of TLR pathways in IgAN pathogenesis. A better understanding of TLR7/9-mediated mucosal immunity may facilitate the development of more precise, mechanism-based therapeutic strategies for IgAN.
